# Damage Control Management of Perforating Pyometra Presenting with Septic Shock after the Return of Spontaneous Circulation

**DOI:** 10.1155/2020/8545232

**Published:** 2020-11-19

**Authors:** Jumpei Takamatsu, Jinkoo Kang, Aya Fukuhara, Tomoya Matsuda, Tomo Ishida

**Affiliations:** Department of Emergency Medicine, Kansai Rosai Hospital, Amagasaki, Japan

## Abstract

**Introduction:**

Perforation of pyometra is often severe but rare. We report a case of pyometra detected on second-look surgery in an elderly patient with life-threatening septic shock and cardiopulmonary arrest before hospital arrival. *Case Presentation*. A 70-year-old woman with cardiopulmonary arrest received adrenaline. Computed tomography revealed ascites, and abdominal paracentesis was performed to identify the cause of cardiopulmonary arrest. The ascitic fluid was purulent, and intraperitoneal infection was identified. Emergency exploratory laparotomy revealed pyometra.

**Conclusion:**

If perforated, pyometra may cause peritonitis and lethal septic shock. Not only gynecologists but also emergency physicians should be aware of this possibility. Moreover, patient education is necessary. In patients with cardiopulmonary arrest, diagnostic abdominal paracentesis should be performed when the sole imaging finding is ascites. Improving outcomes in patients with difficult-to-diagnose pyometra with cardiopulmonary arrest by implementing damage control strategies before hysterectomy is possible.

## 1. Introduction

Pyometra is a rare condition characterized by pus accumulation due to a bacterial infection in the uterine cavity. It is caused by impaired drainage of the uterine cavity, usually due to stenosis and obstruction of the cervix and vagina. Spontaneous perforation in pyometra is rare, and its consequences are often severe, with a mortality rate of 25%–40% [1–3]. Additionally, 50% of imperforate pyometra cases are asymptomatic, which complicates the issue and makes it difficult to ensure survival of these patients.

Pyometra should be considered when patients present with the “classic triad of purulent vaginal discharge, postmenopausal bleeding, and lower abdominal pain” [4]. Physical findings, including fever and an abdominal mass, are often observed in patients with pyometra; however, some patients experience cardiopulmonary arrest in the absence of these.

A high index of suspicion is required to make an early diagnosis and provide early intervention [5]. Management options include total abdominal hysterectomy with bilateral salpingo-oophorectomy, antibiotics targeting anaerobic organisms, and intensive care unit admission [3, 5]. There have been no reports on treatment strategies after resuscitation in cases where findings could not be confirmed after cardiopulmonary arrest.

We present a case of damage control management of perforating pyometra after the return of spontaneous circulation (ROSC) in an elderly woman presenting with septic shock. Intraperitoneal infection was diagnosed by ascites puncture, and she underwent damage control surgery.

## 2. Case Presentation

A 70-year-old woman with a history of uterine fibroids was looking for an emergency hospital with a chief complaint of dysarthria, when an acquaintance found her collapsed. She could barely speak when found, and upon arrival at emergency services by ambulance, she was in cardiopulmonary arrest. Her electrocardiogram waveform showed a pulseless electrical activity. She had not received bystander cardiopulmonary resuscitation (CPR), and ambulance crews started CPR upon arrival on the scene. After the administration of 1 mg adrenaline in the ambulance, the patient's heartbeat resumed. Thirteen minutes passed from the confirmation of cardiac arrest (at arrival at emergency services) to the ROSC. Her bilateral pupil diameter was 4 mm, but spontaneous breathing soon resumed.

**Figure 1:**
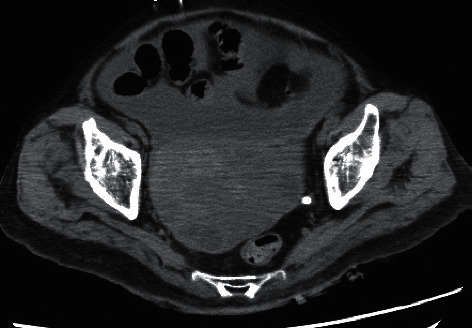
Plain computed tomography (CT) on admission showing ascites accumulation and fluid-containing uterine fibroids.

On arrival, her vital signs were as follows: heart rate, 72 beats/min; blood pressure, 79/35 mmHg; and body temperature, 30.8°C. Assessment of her level of consciousness using the Glasgow Coma Scale revealed eye response of 1, verbal response of 1, and motor response of 1 (E1V1M1), but the bilateral pupil diameter had reduced to 3 mm. Blood gas analysis revealed pH of 6.909, PaCO_2_ of 80.8 mmHg, PaO_2_ of 67.9 mmHg, HCO_3_^−^ level of 17.5 mmol/L, and lactate level of 7.0 mmol/L. On abdominal computed tomography (CT) (Figure 1), we observed ascites accumulation and fluid-containing uterine fibroids of approximately 10 cm in size.

**Figure 2:**
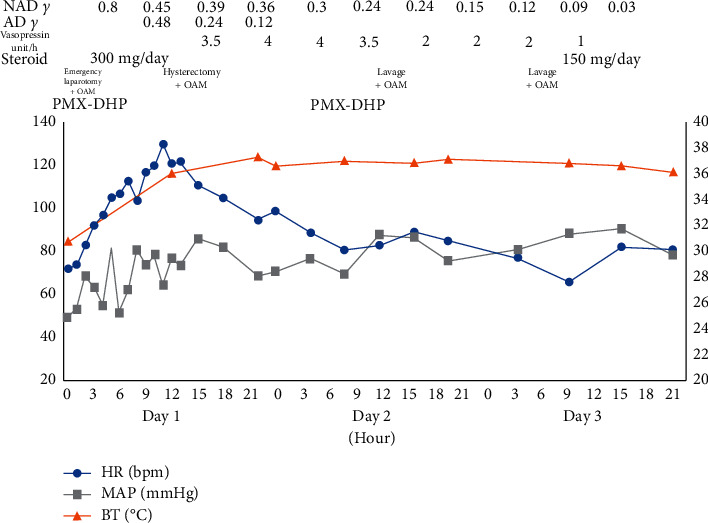
Clinical course after admission. Since the patient was initially in severe septic shock, intensive care was given according to the damage control strategy. As a result, her circulatory dynamics became stable by the end of the day. NAD, noradrenaline; AD, adrenaline; OAM; open abdomen management; PMX-DHP, direct hemoperfusion with polymyxin B-immobilized fibers; HR, heart rate; MAP, mean arterial pressure; BT, body temperature.

**Figure 3:**
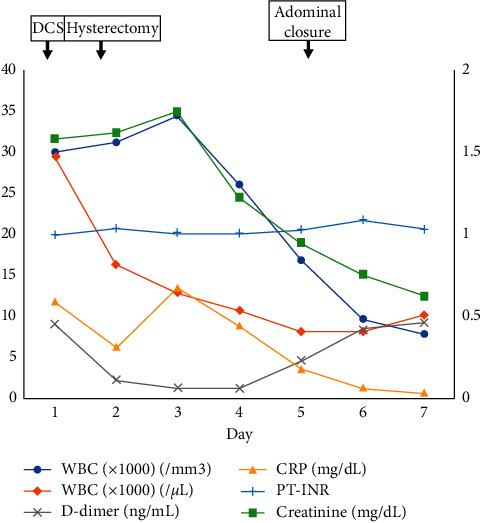
Changes in laboratory data after admission. Inflammatory parameters, renal function, and clotting ability of blood were generally stable within a week. DCS, damage control surgery; WBC, white blood cell; Plt, platelet; CRP, C-reactive protein.

Figure 2 shows the patient's clinical course, and Figure 3 shows her laboratory data after admission. We could not identify the cause of cardiopulmonary arrest; thus, the nature of the ascites detected on CT was assessed. Puncture revealed the ascites to be purulent. We suspected septic shock due to an intraperitoneal infection and decided to perform emergency laparotomy, when a large volume of purulent ascites flowed out. Based on intra-abdominal findings, we speculated that some organs in the pelvis might have been infected, but we could not find the origin in the first-look surgery.

As a damage control strategy, we only performed open abdominal drainage and did not remove the uterus. For temporary abdominal closure, we used open abdomen management (OAM) using the VAC® system (KCI, San Antonio, TX, USA), with polymyxin B-immobilized fiber column direct hemoperfusion (PMX®-DHP, Toray, Tokyo, Japan) immediately after surgery to stabilize the patient's circulatory dynamics.

**Figure 4:**
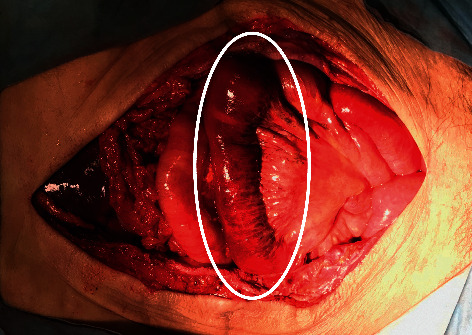
Operative findings on second-look surgery, with a pale small intestine, indicating that it might become ischemic (circle).

Twelve hours after the patient's circulatory dynamics stabilized, we performed second-look surgery and again washed out the intra-abdominal cavity. On re-examining the uterus, we found a pore of approximately 1 cm at the top of the fibroid on the dorsal side and continuous discharge of pus. We judged the cause to be sepsis and immediately removed the uterus, with gauze packing to control oozing from the bottom of the pelvis. The small intestine was ischemic; hence, we again performed OAM for temporary abdominal closure (Figure 4).

We closed the abdomen on day 5 after the first damage control surgery. The patient's vital signs stabilized, and her inflammatory parameters improved and, finally, so did her level of consciousness.

**Figure 5:**
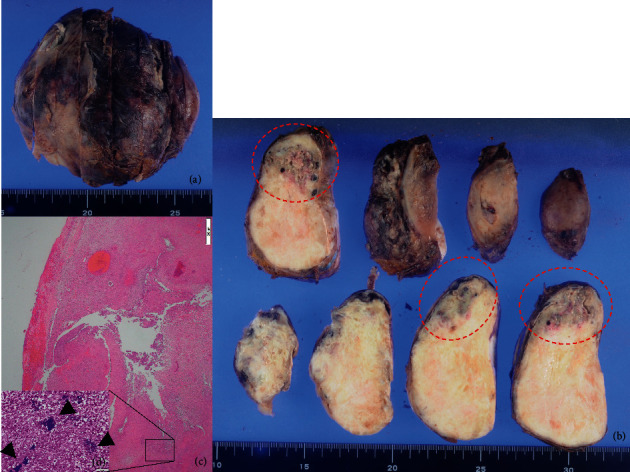
Pathological findings. (a) Appearance of pyometra. (b) A solid white mass of approximately 10 cm in size, and yellowish fragile areas with necrosis or abscess formation in the mass (circles). (c) Histologically, there was no obvious malignancy. There were few positive findings of smooth muscle markers (SMA, Caldesmon) using immunostaining. Deposition of collagen fiber bundles was relatively prominent in the stroma, and a flower bud-like array was recognized. These findings suggested that this mass was a fibroma rather than a leiomyoma. An abscess with massive neutrophil infiltration was present in the mass, and the surrounding serosal surface showed inflammatory cell infiltration with fibrin deposition. (d) A large mass of Gram-positive bacteria (arrowheads) was found inside the abscess.

Pathological findings (Figure 5) included a fibroma with abscess formation, peritonitis, adenomyosis, endometrial polyp, and endometrial stromal nodule of the uterine corpus. Examination of the uterus for pyometra revealed yellowish fragile areas with necrosis or abscess formation (Figures 5(a) and 5(b)). No malignancy was identified on histological examination (Figure 5(c)), suggesting a fibroma with inflammatory cell infiltration. Both blood and ascites cultures were positive for *Peptostreptococcus micros*, an anaerobic bacterium.

Soon after, the patient developed urinary tract infection due to right ureteral calculi, and extracorporeal shock wave lithotripsy was performed. On day 146, she was transferred to another hospital for rehabilitation for hypoxic encephalopathy.

## 3. Discussion

Pyometra may cause peritonitis and lethal septic shock if intraperitoneal perforation occurs. Causes of pyometra include malignant or benign gynecologic tumors, radiation cervicitis, atrophic cervicitis with aging, and intrauterine devices [6–8]. In our patient, pyometra was due to her history of uterine fibroids, which are among the commonest tumors in women, most being followed up.

An aging population suggests that cases of pyometra will increase. Pyometra is usually a chronic disease with a good prognosis, but sometimes it may burst into the peritoneal cavity. In such cases, it may cause generalized peritonitis and has a poor prognosis. Because of the nonspecific symptoms associated with rupture, an accurate diagnosis is often challenging. This can cause a delay in identifying the source of infection, which results in delayed removal of the infection source.

Therefore, not only gynecologists but also emergency physicians should be aware that the perforation of pyometra can be a cause of abdominal pain in elderly women [9]. However, it is also necessary to educate patients about uterine fibroids and about the fact that, in rare cases, they may develop infections that could prove fatal.

As in our case, pyometra is often diagnosed based on findings, including fever, abdominal pain, and abdominal mass. In contrast, there is a report on a patient in whom pyometra could not be confirmed from findings, including those after resuscitation [7]. A common treatment for pyometra is transvaginal uterine washing; however, in the case of perforation, intraperitoneal irrigation drainage by laparotomy, total hysterectomy, and superior uterotomy are preferred [2].

A report has suggested that removal of uterine necrosis, closure of the perforation, and transabdominal uterine drainage without total uterine removal can save the patient's life [10], but, in most previous cases, total hysterectomy has been selected because of its better outcome [3]. Currently, no standard treatment strategy has been reported, and there are no clear criteria to select a damage control strategy for atraumatic patients. However, in the most severe instances, the patient may benefit from a damage control strategy [11]. In our case, we were unaware of the fact that her primary care doctor had previously detected a pyometra. It took over 10 minutes to resuscitate the patient; thus, we thought that it would be difficult to save her life, especially that her hemodynamics on arrival were unstable. Therefore, we judged that open abdominal drainage was sufficient for source control.

We suggest that if even a slight improvement in the neurological prognosis is anticipated—similar to that observed in our patient—aggressive treatment, including searching for the cause and surgery, should be considered. Abdominal echography or CT should be performed to identify the cause, followed by ascites puncture when ascites is confirmed. If ascites puncture identifies an intraperitoneal infection, abscess drainage by opening is a reasonable strategy for source control [12] and can be performed as a damage control surgery.

Spending time searching for the cause of the intraperitoneal infection after resuscitation and continuing with primary curative surgery will likely result in failure of circulatory dynamics. After stabilizing the patient's circulatory dynamics in the intensive care unit, it is possible to identify the cause by examining the intra-abdominal cavity in second-look surgery. Our patient survived because of hysterectomy performed to treat pyometra, which was determined to be the cause of the life-threatening septic shock at second-look surgery. If a damage control strategy is undertaken, as reported here, outcomes of patients with difficult-to-diagnose pyometra who experience cardiopulmonary arrest may improve.

## Data Availability

The data used to support this study are restricted to protect patient privacy.
